# Child health and the implementation of Community and District-management Empowerment for Scale-up (CODES) in Uganda: a randomised controlled trial

**DOI:** 10.1136/bmjgh-2021-006084

**Published:** 2021-06-08

**Authors:** Peter Waiswa, Flavia Mpanga, Danstan Bagenda, Rornald Muhumuza Kananura, Thomas O’Connell, Dorcus Kiwanuka Henriksson, Theresa Diaz, Florence Ayebare, Anne Ruhweza Katahoire, Eric Ssegujja, Anthony Mbonye, Stefan Swartling Peterson

**Affiliations:** 1 Department of Health Policy Planning and Management, Makerere University School of Public Health, Kampala, Uganda; 2 Makerere University Centre of Excellence for Maternal Newborn & Child Health, Makerere University School of Public Health, Kampala, Uganda; 3 Global Public Health, Karolinska Institute, Stockholm, Sweden; 4 Busoga Health Forum, Jinja, Uganda; 5 UNICEF Uganda Country Office, Kampala, Uganda; 6 University of Nebraska Medical Center, College of Medicine, Omaha, Nebraska, USA; 7 Department of International Development, London School of Economics and Political Science, London, UK; 8 World Health Organization, Geneve, Switzerland; 9 Department of Maternal, Newborn, Child and Adolescent Health and Ageing, World Health Organizations, Geneva, Switzerland; 10 Child Health and Development Centre, Makerere University, Kampala, Uganda; 11 School of Public Health, College of Health Sciences, Makerere University, Kampala, Uganda; 12 Programme Division, Health Section, United Nations Children's Fund, New York, New York, USA

**Keywords:** child health, health systems, cluster randomised trial, public health

## Abstract

**Introduction:**

Uganda’s district-level administrative units buttress the public healthcare system. In many districts, however, local capacity is incommensurate with that required to plan and implement quality health interventions. This study investigates how a district management strategy informed by local data and community dialogue influences health services.

**Methods:**

A 3-year randomised controlled trial (RCT) comprised of 16 Ugandan districts tested a management approach, Community and District-management Empowerment for Scale-up (CODES). Eight districts were randomly selected for each of the intervention and comparison areas. The approach relies on a customised set of data-driven diagnostic tools to identify and resolve health system bottlenecks. Using a difference-in-differences approach, the authors performed an intention-to-treat analysis of protective, preventive and curative practices for malaria, pneumonia and diarrhoea among children aged 5 and younger.

**Results:**

Intervention districts reported significant net increases in the treatment of malaria (+23%), pneumonia (+19%) and diarrhoea (+13%) and improved stool disposal (+10%). Coverage rates for immunisation and vitamin A consumption saw similar improvements. By engaging communities and district managers in a common quest to solve local bottlenecks, CODES fostered demand for health services. However, limited fiscal space-constrained district managers’ ability to implement solutions identified through CODES.

**Conclusion:**

Data-driven district management interventions can positively impact child health outcomes, with clinically significant improvements in the treatment of malaria, pneumonia and diarrhoea as well as stool disposal. The findings recommend the model’s suitability for health systems strengthening in Uganda and other decentralised contexts.

**Trial registration number:**

ISRCTN15705788.

**Summary box**

**What is already known?**

- Four factors constrain performance to improve child health: a lack of supportive policies; an inability to prioritise high-impact, evidence-based interventions; weak or broken supply chains for curative and preventative commodities and the absence of community-based health promotion and care.
- Limited managerial capacity hinders the planning and management of decentralised health services, and communities lack the means to demand or demonstrate accountability for even the most basic health services.

**What are the new findings?**

- We provide evidence on how the data-driven decision-making at different levels of implementation improved access to the required preventive and curative services for children in a decentralised resource-limited setting.

**What do the new findings imply?**

- The findings show that it is possible to create a system for district health managers to use data, act on these data and provide quantitative proof of impact on health outcomes. However, to be effective, it is important to bridge the limited fiscal space that the district managers have to enable them to implement the identified solutions.

## Background

Global progress in reducing under-five mortality remains uneven.^1–5^ In sub-Saharan Africa and other regions plagued with persistently high rates of child mortality, four factors constrain performance: a lack of supportive policies; an inability to prioritise high-impact, evidence-based interventions; inadequate skilled professional; weak or broken supply chains for curative and preventative commodities and the absence of community-based health promotion and care.^3–5^ Compounding the complex mix of challenges is the recent trend towards decentralisation. Central governments are devolving select managerial functions to district-level administrators who are often unprepared and ill-equipped to assume responsibility for critical health services.^6–10^ As limited managerial capacity hinders the planning and management of decentralised health services, dissatisfaction intensifies among health system users and workers.^11–13^ Circumstances deteriorate further still when communities lack the means to demand or demonstrate accountability for even the most basic health services.^10^ In Uganda, a multiyear initiative called Community and District-management Empowerment for Scale-up (CODES) aimed to enhance the government’s ability to combat diarrhoea, pneumonia and malaria—three of the country’s leading causes of child mortality. Building on the analytic framework pioneered by Tanahashi, CODES contributed to the identification of the source and potential remedy for major health system bottlenecks.^14^ Like Tanahashi’s diagnostic model, CODES emphasised ‘effective coverage’, meaning the quantity of quality interventions needed to achieve the desired health impact.^15^ The CODES model identified four determinants of effective service coverage: the enabling environment, supply, demand and the quality of services. Intended to accommodate diverse health system delivery platforms, CODES strived to help managers target resources towards context-appropriate solutions.^16^

Applying bottleneck analysis at district level to key child health interventions,^16 17^ together with community dialogue^18^ for social accountability, we designed an randomised controlled trial (RCT) to test the hypothesis that intervention districts would improve the coverage and quality of key protective, preventive and curative indicators for pneumonia, diarrhoea and malaria^19^ compared with comparison districts.

## Methods

### The CODES intervention package

CODES was designed to diagnose and resolve health system bottlenecks, primarily the challenges related to the district’s management of local health services. The implementation of the intervention was carried out within the district health structure by selected partners (online supplemental table 1) under the management of UNICEF and Ministry of Health. The implementation followed a predesigned and static theory of change or logic framework (online supplemental material 1). The management intervention involved three mutually reinforcing pillars:

10.1136/bmjgh-2021-006084.supp5 Supplementary data

10.1136/bmjgh-2021-006084.supp1 Supplementary data

pillar 1 consisted of collating, analysing and applying programme and survey data. In each intervention district, the authors conducted three separate annual rounds of lot quality assurance sampling (LQAS)^20^ to identify underperforming indicators and underserved populations. Intervention districts used the data to prioritise bottlenecks and identify solutions. The solutions were costed and incorporated in annual district work plans. Districts submitted their completed work plans to relevant bodies for approval, as per normal practice.^21^ To facilitate the implementation of locally identified solutions, UNICEF supplemented district budgets with a ‘bottleneck fund’ of US$10 000 per district per year.

**Table 1:** Percentage of coverage, difference in differences and ORs for quality protective indicators in intervention and comparison districts areas at the baseline and end line

Measure	Baseline			Endline			DID change in %			OR (95% CI)**	P value**
	Intervention	Comparison	P value*	Intervention	Comparison	P value†	DID	95 % CI	P value*		
Recommended use of vitamin A; children 12–23 months	n=665 68.9 (64.6–72.8)	n=850 61.7 (52.2–70.3)	0.13	n=760 74.6 (69.3–79.3)	n=757 72.3 (67.5–76.5)	0.47	−4.9	−16.5, 6.7	0.383	1.1 (0.8–1.5)	0.54
Recommended use of vitamin A; children 6–11 months	n=664 66.4 (60.7–71.7)	n=848 68.9 (60.4–75.6)	0.65	n=762 74.4 (69.8–78.6)	n=760 67.1 (61.7–72.1)	0.037	+9.4	−1.8,20.6	0.095	1.4 (1.1–1.9)	0.016
Recommended exclusive breastfeeding children 0–5 months	n=665 62.8 (55.8–69.3)	n=850 69.3 (64.7–73.6)	0.11	n=762 66.4 (60.4–71.9)	n=760 68.6 (63.5–73.2)	0.69	+4.4	−6.4,+51.1	0.398	1.1 (0.7–1.6)	0.65
Mothers of 0–5 months old at least 4 ANC visits—ANC card	n=665 23.5 (19.5–27.9)	n=852 25.5 (21.1–30.4)	0.52	n=759 23.6 (19.1–28.7)	n=760 22.9 (18.9–27.5)	0.89	+2.7	−6.5,11.9	0.541	0.9 (0.6–1.3)	0.62
Mothers of 0–5 months old who gave birth in health facility with assistance from a skilled birth attendant during last pregnancy	n=665 61.4 (54.7–67.6)	n=852 67.1 (60.9–72.8)	0.20	n=759 67.3 (62.6–71.7)	n=760 73.2 (67.5–78.1)	0.14	−0.05	−10.1,10.0	0.992	0.9 (0.7–1.1)	0.23
Mothers of 0–5 months old who were tested for HIV and received their test results during last pregnancy	n=665 75.5 (68.9–81.0)	n=852 81.9 (77.2–85.9)	0.08	n=759 78.5 (73.1–83.1)	n=760 81.6 (76.3–85.9)	0.52	+3.4	−10.2,16.5	0.603	0.9 (0.5–1.7)	0.82

*Cluster design adjusted.

†End line p value cluster design adjusted ANCOVA, baseline is cluster design adjusted.

‡OR estimates are based on a GEE ANCOVA baseline district indicator average and cluster design adjusted analysis.

ANCOVA, analysis of covariance; DID, difference-in-differences; GEE, generalized estimating equation.

Pillar 2 involved regularly reviewing and, where necessary, supporting the implementation of district work plans. District Health Management Teams were encouraged to monitor implementation regularly, initiating quality improvement efforts within each planning cycle. District scorecards and a mentorship programme facilitated interdistrict learning.

Pillar 3 aimed to stimulate demand for services through community engagement. In each catchment area, 70–100 community members joined local leaders to discuss LQAS survey findings, which were summarised in citizen report cards (see online supplemental figure 1). The participatory forums provided community members and healthcare workers with a unique opportunity to build consensus on priority problems and solutions. At the end of the community dialogue, participants prioritised their proposed actions in ‘community contracts’. Community volunteers then monitored and reported on implementation using ‘U-Report’, an SMS-based platforms’ local radio stations lent additional momentum to the collective effort, promoting public demand for health services and advocating for the speedy implementation of community contracts.^19^

10.1136/bmjgh-2021-006084.supp2 Supplementary data

A series of sensitisation meetings introduced CODES to participating districts. Three implementing partners facilitated the meetings: Child Fund International (CFI) and the Liverpool School of Tropical Medicine (LSTM), both focused on the supply side of the intervention and the Advocates Coalition for Development (ACODE), which concentrated on the demand side.

Throughout the RCT, district data analysis, district management and community contacts remained unchanged in comparison districts. The results of the LQAS surveys in comparison districts were delivered in reports that had a tabular format.

### The bottleneck analysis

Adapted from Tanahashi,^14 16^ the analytic model for health system bottlenecks was applied to assesses six factors when appraising the effective coverage of select health services: (1) availability of essential commodities, (2) availability of human resources, (3) accessibility of distribution points, (4) initial utilisation of the intervention, (5) continued usage and (6) the quality of the intervention.^19^ The bottleneck analysis is normally presented as a graph that cascades across categories such as supply, demand and quality. Each determinant is influenced by its predecessor in a manner that indicates a potential ‘bottleneck’ to be addressed. For the purposes of the CODES RCT, the authors selected tracer interventions from across the spectrum of ‘protect’, ‘prevent’ and ‘treat’.^16 22^ While the Excel-based bottleneck tool used for CODES did not explicitly capture indicators for policies, social norms, budgets and related coverage determinants, these were considered when analysing the root causes of each bottleneck.

### Study design and district randomisation

After an initial assessment of Uganda’s 111 districts, the RCT targeted 16 high-mortality districts, and eight were randomly selected for each intervention and comparison. A sampling frame of 25 districts was selected purposively by UNICEF based on the absolute number of deaths expected for districts, which was obtained by applying regional DHS Infant and child mortality data to the projected district population of children under five. Details of the trial protocol and early implementation are published elsewhere.^4 9 10 19^

Prior to randomisation, the authors matched districts based on an index of 20 CHERG-based child survival indicators.^23^ The composite index was weighted based on each indicator’s: level of coverage in the district; impact on child mortality^23^ and the proportion of total mortality attributed to a specific cause.^23^ Thus, for each district, the index was calculated by summing all 20 child survival indicators and assigning a weight based on the three components. Since the districts in the sampling frame were quite heterogeneous, there was a need to stratify them in order to ensure that randomisation into intervention and control arms would occur within strata that were relatively well ‘balanced’ for fair comparison on some key factors associated with heterogeneity—that is, each randomly selected intervention district would have a corresponding randomly selected control district selected from the same strata—(ie, matched within the strata). Some factors that were in consideration for stratifying the sampling frame (online supplemental figure 2):

10.1136/bmjgh-2021-006084.supp3 Supplementary data

1. Whether this was parent (old) or child (new) district—given that new districts may not have been adequately constituted with human resources and infrastructure. Therefore, controlling for this as best as possible was critical given the intervention’s nature and outcomes of interest.
2. Stratification on the current coverage of key child survival indicators pertaining: to do this, a composite Index was formed using available key child survival indicators of pneumonia, diarrhoea, malaria for each of the 25 districts. The Indicator Composite Index was created as follows: inputting current level of coverage indicators for 21 Prevention Protect Treat child survival indicators for Pneumonia, Diarrhoea, Malaria; impact on mortality of each of the indicators; the proportion of total mortality attributed to a cause. For each district, a composite index was calculated by summing over all the available key child survival indicators the product of these three components. A threshold for this index was used to divide into the sampling frame further in those districts below or above the median threshold.

**Figure 1:**
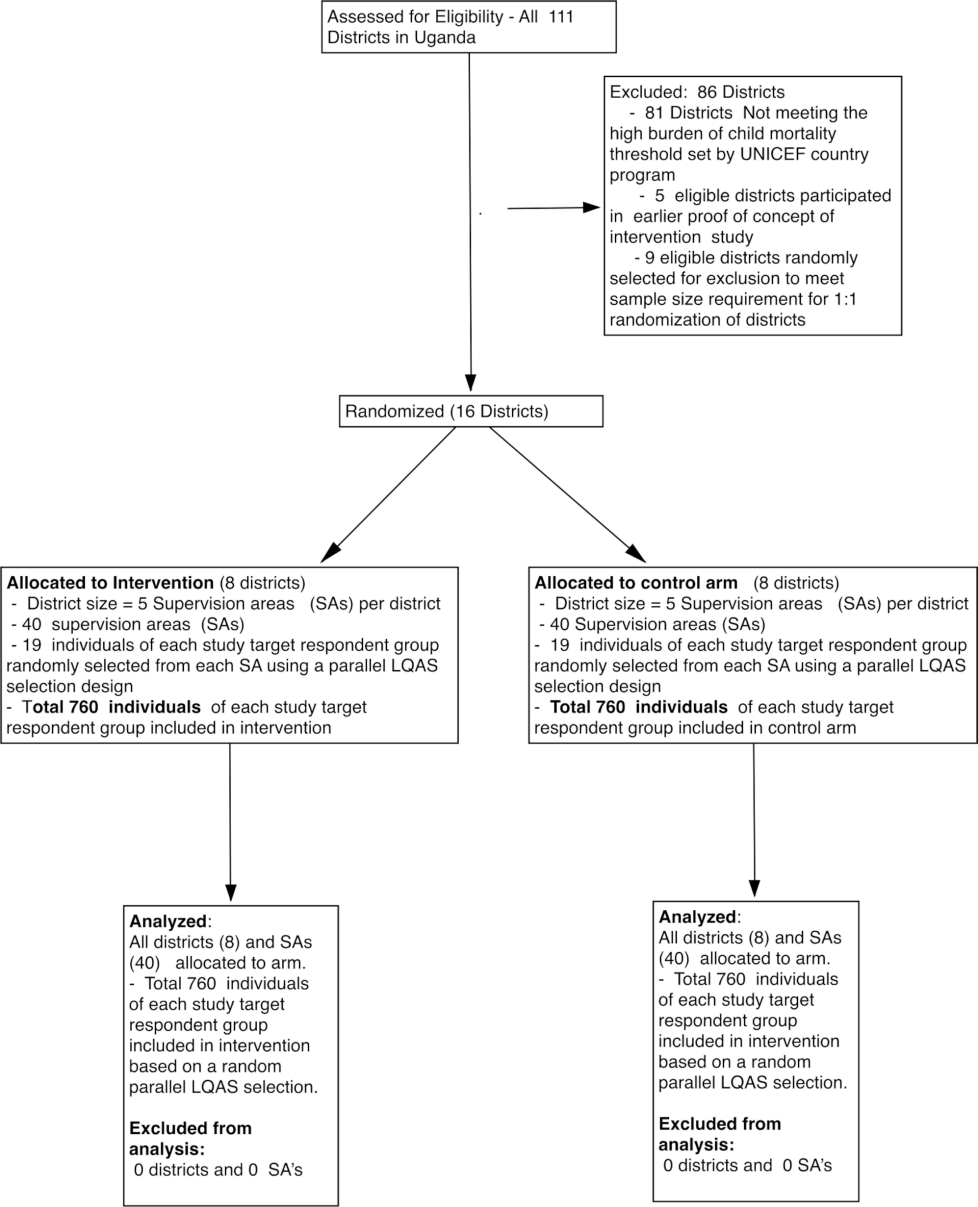
Trial profile. LQAS, lot quality assurance sampling; SA, sepervision areas.

Within the strata (online supplemental figure 2), we randomised districts to intervention: control in 1:1 ratio of intervention going for a homogenous match with respect to the factor indicated. After applying the eligibility criteria, the authors randomly selected eight pairs from the remaining districts. A coin toss determined which of the two districts from each pair would be targeted by the CODES intervention (figure 1). Makerere University remained independent of the study and conducted the randomisation. Descriptive district data are presented in online supplemental table 2.

10.1136/bmjgh-2021-006084.supp8 Supplementary data

The study districts were not aware of their allocation status to intervention or comparison group.

### Sample size calculations

Sample size computations were based on the baseline for each coverage and quality indicator (see list of key primary outcome indicators) obtained from wave 0 districts (CODES pilot districts) (online supplemental figure 3) and Uganda Demographic Health Surveys 2011 estimates. We assumed 80% power of observing difference; differences of 25%–30% (regarded to be of public health significance) between intervention and control and a 5% significance level. We accounted for a design effect (intracluster correlation (ICC) of cluster randomised design—worst-case ICC=0.20). Details on sample size calculation can be found in the implementation protocol.^19^

10.1136/bmjgh-2021-006084.supp4 Supplementary data

### Outcomes

As indicated in the implementation logic framework (online supplemental material 1), the primary outcome measures were protective, preventive and curative indicators for the effective coverage of pneumonia, diarrhoea and malaria interventions, as described below and in the protocol paper.^19^

*Protective coverage:* exclusive breastfeeding for 6 months and adherence to the recommended schedule of vitamin A supplementation.

*Preventive indicators:* full immunisation (based on standard age-specific vaccination) within the first year of life; the standard diphtheria-pertussis-tetanus (DPT) 3 indicator; use of long-lasting insecticide-treated nets; improved water and sanitation and handwashing with soap.

*Curative indicators:* appropriate case management for malaria, pneumonia and diarrhoea symptoms.

We also evaluated secondary outcomes, including the 2-week prevalence of pneumonia, diarrhoea and malaria symptoms among the target under-5-year age-group.^19^ Additional effort was made to document immediate management outcomes such as annual reports that prioritised bottlenecks for pneumonia, diarrhoea and malaria. The study was not designed to measure changes in mortality.

### Data collection and analysis

The data analysis in this manuscript is based on the primary data at baseline between December 2013 and January 2014 and end line data collected between June and July 2016. The data collection relied on LQAS household surveys in both intervention and comparison districts at the baseline, midterm (2 years later and in intervention districts only) and end line, approximately 33 months after the baseline. The participating 16 districts were divided into five supervision areas. Information from the Uganda Bureau of Statistics^24^ facilitated the random selection of 19 villages from each of the five supervision areas, based on the number of households with probability proportionate to size. Assisted by UNICEF, the Ministry of Health (MOH) and implementing partners, the districts were trained on lot quality assurance sampling (LSQA) data collection methodology and were responsible for data collection.

A total of 760 individuals from each target group were sampled in both the intervention and control arms, 95 per district in each of the five areas. The household LQAS surveys contained seven target population groups: mothers of children <6 months; mothers of children 6–11 months; mothers of children 12–23 months; mothers of children <5 years; mothers of children <5 years with diarrhoea in the last 2 weeks; mothers of children <5 years with acute respiratory tract infections in the last 2 weeks and mothers of children <5 years with fever in the last 2 weeks. In each village, a random reference household was selected. The next nearest door to this household determined the first and subsequent household from which one interviewee was selected, with a maximum of one respondent per household.

Adherence to the planned CODES interventions was assessed against reports from the implementing partners (CFI/LSTM and ACODE). Changes in management behaviour were measured through data obtained from participant observation, in-depth interviews and an analysis of relevant district documents, including district health plans and implementation reports.^9 10^ To evaluate community participation and demand-side behaviour, we collated data from SMS surveys, LQAS surveys and focus group discussions.^25^

Primary analysis relied on an intent-to-treat approach. The authors conducted a difference-of-differences analysis of indicators between the baseline and end line for the intervention and control districts, adjusting for the cluster randomised design^26 27^ and using techniques that included a cluster-specific and population-averaged approach to longitudinal data analyses,^28 29^ generalised estimating equations and random-effects logistic regression with analysis of covariance^30^ as well as cluster-adjusted χ^2^ tests.^31^ ORs and CIs were calculated and are reported here.

### Costing the CODES project

Based on the eight wave one districts, we conducted a costing study to estimate the cost of scaling up the CODES project to all districts in Uganda. Three scenarios were considered for costing of the scale-up of CODES activities to 115 districts (excluding Kampala). A full description of what these scenarios entail will be published elsewhere. In scenario 1, scale-up of the CODES package includes all activities of the CODES package as was implemented during the wave 1 pilot phase, excluding costs for operational costs but including minimal costs for technical assistance. In scenario 2, scale-up of CODES package includes ‘selected activities’ which are considered critical to strengthening district health systems through improved management and supervision. On the demand side, we included: community dialogues, national advocacy campaigns and other materials for dialogues (citizen report cards, poster illustrations and hot-line cards). On the supply side, we included: Health Facility Quality of Care Assessments, Bottleneck, Casual and Management Analysis workshop, work plan development and routine supervision. We considered the smallest ‘incremental package’ for scenario 3, which includes only activities that are unique to CODES and are not being routinely undertaken already. On the demand side, we considered community dialogues and provision of materials to support community dialogues. On the supply side, we only considered: bottleneck analysis, Causal Analysis and Management Analysis workshops.

### Study ethics and registration

The Uganda National Council for Science and Technology granted ethical clearance for the study (Ref: SS2548). All participating districts were implementing the Uganda Child Survival Strategy, in accordance with the Health Sector Development Plan.^32^ After explaining the study’s objectives and procedures, the authors obtained the informed consent of each participating individual. Confidentiality was maintained throughout the study. A Study Steering Committee chaired by the Ministry of Health convened the study team quarterly to facilitate policy linkages.

### Role of the funding source

Both the Bill & Melinda Gates Foundation and the UNICEF Fund for USA were involved in periodic progress reviews. Neither funding source influenced the design of the RCT nor any aspect of the data collection, analysis or interpretation of the study’s findings. The corresponding author had full access to all the data in the study and responsible for submitting the study’s findings for publication.

### Patient and public involvement statement

We involved the district health team, patients, community groups and national and international stakeholders in the design of the project. Throughout implementation, the patients were consulted through the U-reporting platform for their feedback on service delivery. The district health team was responsible for collecting data and disseminating results that informed their planning and implementation. We also had a national advisory committee whose role was to regularly guide the implementation, diffusion and sustainability of the implementation strategies.

## Results

Figure 1 shows the trial profile of the intervention. Sociodemographic characteristics of the samples are detailed in online supplemental table 2.

### The intervention dose delivered

Online supplemental table 2 shows the intervention dose delivered in the trial. All interventions were delivered as planned in terms of numbers. However, the table also shows that most activities were on the supply side, especially at the district level and in health facilities, with relatively few on the demand side through community dialogues.

### Management outcomes

All intervention districts developed work plans that prioritised bottlenecks in managing pneumonia, diarrhoea and malaria. Each intervention district received US$10 000 to supplement existing budgets. Even with the supplemental funding, many participating districts lacked the funds to implement prioritised interventions.^33^ Variations in management behaviours during implementation were observed across interventions and sites. We observed that some districts were more receptive than others, especially for the newly formed districts compared with those that had been in existence for some years (new vs old). It also emerged that districts whose management teams were fully constituted performed better at adoption compared with those with partial composition or ones with managers not in substantive positions (acting vs substantive). It also emerged that districts with longer exposure (wave 0 and wave 1) performed better than those exposed to the intervention only during wave 1 (wave 1 vs wave 0+wave 1). We also observed differences in the choice of interventions for funding using the slush fund to supplement existing budgets.

### Effect on protective coverage indicators

Table 1 shows the effect of CODES on the indicators for protective interventions. Positive, statistically significant effects were registered for the recommended use of vitamin A 9+9·4%) among children 6–11 months. The effects on vitamin A use in the older child and on exclusive breastfeeding were not statistically significant.

### Effects on preventive coverage indicators

#### Immunisation and use of Insecticide-treated bed nets (ITNs)

Table 2 shows the effects on measures for preventive coverage. There were positive, not statistically significant effects on DPT3 (net improvement 7·8%) and full immunisation (net improvement 7·3%) coverage. Overall, however, coverage levels remained below national and WHO targets. Effective bed net usage remained largely unchanged following the intervention. However, the percentage of households with drinking water from safe sources appears to have increased by a net of 7·4% (CI −1·6 to 16·5) in favour of intervention districts. Net safe stool disposal increased significantly, with a rise of +10·4% (CI 4·9 to 15·9).

**Table 2:** Percentage of coverage and difference in differences for quality preventative indicators in intervention and comparison districts at the baseline and end line

Measure	Baseline			Endline			DID change in %			OR (95% CI)**	P value**
	Intervention	Comparison	P value*	Intervention	Comparison	P value†	DID	95 % CI	P value*		
Children 0–59 months											
That used a bed net the night before	n=663 63.7 (55.0–70.9)	n=851 64.0 (56.1–71.3)	0.94	n=761 65.4 (59.1–71.3)	n=759 69.4 (64.8–73.7)	0.51	−3.6	−29.2,22.0	0.769	0.8 (0.5–1.4)	0.48
Whose homes used an improved drinking water source (piped water; protected well or borehole)	n=663 75.4 (64.4–83.9)	n=851 80.3 (74.8–84.8)	0.36	n=761 83.4 (75.1–89.4)	n=757 80.9 (74.6–85.8)	0.75	+7.4	−1.6,16.5	0.101	1.8 (1.3–2.7)	0.0018
Whose household used/had access to an improved latrine	n=663 2.9 (1.3–6.1)	n=851 2.1 (1.0–4.4)	0.57	n=761 1.5 (0.7–2.8)	n=757 1.7 (0.8–3.7)	0.67	−1.0	−4.6,2.5	0.547	0.9 (0.4–2.2)	0.79
Household where last stool was disposed of in latrine (ie, safely)	n=663 73.8 (64.8–81.1)	n=851 87.0 (82.6–90.4)	0·0018	n=761 81.9 (74.2–87.6)	n=757 84.7 (79.5–88.8)	0.69	+10.4	4.9,15.9	0.001	1.6 (1.1–2.2)	0.016
Children 12–23 months											
Immunisation DPT 3 (card verified)	n=665 40.0 (34.4–45.9)	n=850 45.8 (40.2–51.4)	0.15	n=760 48.6 (42.0–55.2)	n=757 46.5 (41.3–51.8)	0.73	+7.8	−6.1,21.8	0.25	1.2 (0.7–1.9)	0.60
With full immunisation card verified vaccinated for BCG, polio4+, DPT3 +and measles	n=665 13.2 (10.0–17.3)	n=850 19.4 (15.3–24.3)	0.031	n=760 22.4 (17.7–27.8)	n=757 21.3 (17.2–26.1)	0.80	+7.3	−3.6,18.1	0.17	1.3 (0.7–2.2)	0.44

*Cluster design adjusted p-values.

†End line p-value cluster design adjusted, ANCOVA p-values, baseline is cluster design adjusted.

ANCOVA, analysis of covariance; BCG, Bacille Calmette-Guérin; DPT3, diphtheria-pertussis-tetanus.

### Effect on coverage indicators for curative intervention

Table 3 summarises the net effect of the implementation by comparing the two-study arms baseline and end-line results. Recommended treatment for malaria increased by 6·7% in intervention districts and fell by 16·6% in comparison districts, resulting in a net change of 23·3% (CI 9·1 to 37·5). Adherence to the recommended treatment of pneumonia symptoms increased by 11% in intervention districts and declined by 8% in comparison areas, indicating a statistically significant 19·2% (CI 7·9 to 30·6) intervention effect. Treatment for diarrhoea showed a 13·2% (CI 5·6 to 20·7) positive intervention effect with higher utilisation of zinc and oral rehydration solutions (ORS) in intervention areas. The effects of the intervention on the 2-week prevalence of symptoms of malaria (fever), pneumonia (cough) and diarrhoea were insignificant at 95% CI.

**Table 3:** Percentage of the coverage and difference in differences for quality treatment indicators in intervention and comparison districts areas at the baseline and end line

Measure	Baseline			Endline			DID change in %			OR (95% CI)**	P value**
	Intervention	Comparison	P value*	Intervention	Comparison	P value†	DID	95 % CI	P value*		
Children 0–59 months											
With confirmed malaria recommended treatment for malaria	n=247 36.0 (28.5–44.4)	n=382 51.8 (44.8–58.8)	0·0041	n=368 42.7 (33.5–52.3)	n=361 35.2 (28.9–42.1)	0.14	+23.3	9.1,37.5	0.003	1.4 (1.01–2.0)	0.047
With confirmed ‘pneumonia’ recommended treatment for ‘pneumonia’	n=236 5.9 (3.1–11.2)	n=325 18.5 (12.8–25.9)	0·0014	n=299 17.4 (11.5–25.4)	n=215 10.7 (7.2–15.7)	0.15	+19.2	7.9,30.6	0.003	2.1 (1.02–4.1)	0.042
With diarrhoea (3+watery stool same day or blood criteria) recommended treatment for diarrhoea (Zn +ORS first day for duration)	n=661 0.0 (0.0–0.2)	n=851 0.2 (0.1–1.0)	0.22	n=758 5.0 (3.5–7.1)	n=757 3.3 (2.0–5.4)	0.16	+1.9	−0.6,4.5	0.13	1.3 (0.7–2.1)	0.40
With diarrhoea (3+watery stool same day or blood criteria) treated for diarrhoea (any utilisation Zn +ORS)	n=661 5.9 (3.1–11.1)	n=851 12.2 (8.5–17.2)	0.049	n=758 25.3 (20.3–31.1)	n=757 18.5 (13.4–25.0)	0.071	+13.2	+5.6,+20.7	0.002	1.7 (1.2–2.6)	0.0079

*Cluster design adjusted p-values.

†End line p-value cluster design adjusted, ANCOVA p-values; baseline is cluster design adjusted.

‡

ANCOVA, analysis of covariance; ORS, oral rehydration solutions.

### Overall effects

**Figure 2:**
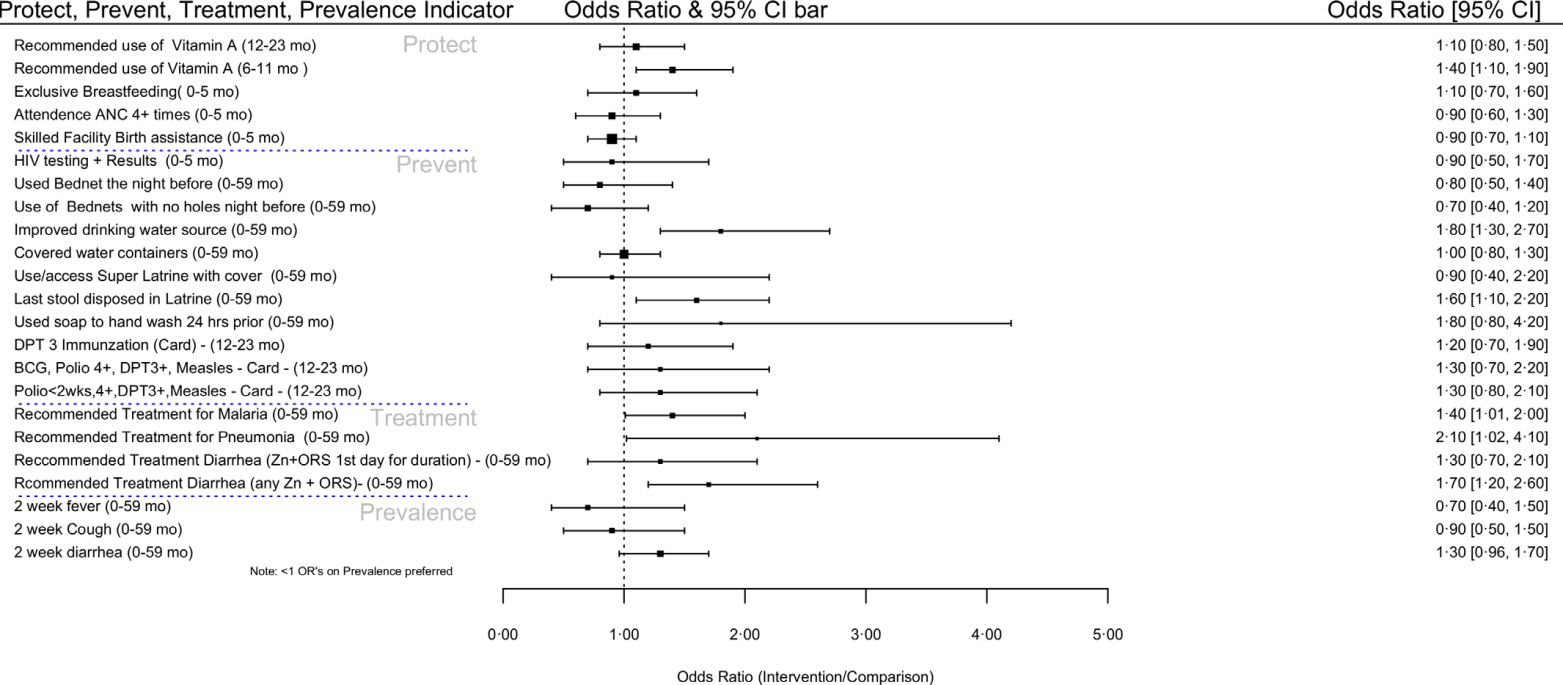
Forest plot summarising the differences in the results between the CODES intervention and control areas. CODES, Community and District-management Empowerment for Scale-up.

Figure 2 summarises the overall results of the CODES study. With the exception of the curative management of malaria, pneumonia and diarrhoea whose odds were higher in the intervention areas, other results reveal insignificant differences between the intervention and control area at 95% CI.

### Cost of scaling up the CODES project

The total cost of all CODES activities implemented in the eight districts in 2014 was US$1 929 014, and it was US$ 1 280 385 in 2015 and US$ 1 221 159 in 2016. Findings of the analysis show that technical assistance accounted for the largest proportion of the total costs, that is, 60% in 2014, 62% in 2015 and 43% in 2016. Operational costs took up the second largest share of total costs, accounting for 20%, 17% and 27% of total costs in 2014, 2015 and 2016, respectively, while demand-side interventions took up 5%, 10% and 15% and supply-side interventions took up 15%, 11% and 15% of total costs, in 2014, 2015 and 2016, respectively. Scenario 1 (the full package) generates the highest total annual scale-up cost of US$5 893 389. Scenario 2 has a total annual scale-up cost of US$2 066 110. Scenario 3 has a total annual scale-up cost of US$661 783.

## Discussion

Health managers in all intervention districts applied CODES tools to generate local data, diagnose health system bottlenecks and prioritise solutions.^9 10^ Relative to comparison districts, intervention districts showed both clinically and statistically significant improvements in the management of malaria, pneumonia and diarrhoea symptoms among children of 0–5 years, while there were generally smaller, nonsignificant improvements in protective and preventive coverage indicators. District health managers reported that CODES provided a context-specific methodology for prioritising interventions. However, constraints in the policy and fiscal space open to district health managers impeded the implementation of the locally identified solutions.^9 10^ Similar results were found in Nigeria where despite prioritisation of identified bottlenecks at subnational level, effective implementation called for central government oversight especially those bottlenecks that could not be addressed at lower level.^34^ Comparing across districts leadership and managerial capacity proved crucial for the adoption and successful implementation of childhood interventions.^9^

The study findings have several implications. First, while it is important to reinforce districts’ technical capacity for evidence-based management, decentralisation requires that interventions place equal emphasis on both local managers and local policymakers. Second, evidence is not enough—district managers need the requisite fiscal and decision-making space to design and implement bespoke district work plans.^33^ This finding compares well with results from Ghana and |Tanzania, which revealed that financial barriers were one of the major bottlenecks impeding success in combination with quality coverage.^35 36^ Furthermore, fiscal space alone may prove insufficient, unless district decision-makers value the political benefits of evidence-informed planning.^37^

The implementation of CODES demonstrates that a data-driven deliberate effort to strengthen district managers’ ability to identify, prioritise and resolve health system bottlenecks contributes to health outcomes. The study also underscores the need to identify which level of government is best positioned to activate particular types of solutions. For instance, the solution to a district’s overall lack of bednets, vaccines and drugs likely lies at the national level. A misalignment in the administrative levels between problems and solutions limits the potential impact of localised community action. In such instances, mechanisms must be found to transmit locally identified solutions across the various layers of health system management to the level empowered to solve the problem.^38^

In the CODES study, all intervention districts prioritised improvements in the coverage of treatment for malaria, pneumonia and diarrhoea in their workplans. These areas also saw the highest intervention effects.^39^ However, the coverage improvements in intervention districts stand out in sharp contrast to the deteriorating or stagnant levels of coverage reported in comparison areas, resulting from flat or declining levels of public health financing over the study period^40^ and worsening management capacity. Indeed, we are aware that over the study period, planning and supervision support to districts by the ministry of health was limited and irregular, which must have particularly affected comparison districts. Therefore, it is clear that scaling the gains achieved through CODES would likely require an increase in Uganda’s overall health expenditure, in addition to continued efforts to address public system shortfalls in management, human resources and medicines.^41^

On the demand side, we did not manage to catalyse a large number of community dialogues (17 per intervention district), keeping levels of population coverage and the ‘intervention dose’ low (see online supplemental table 4).^10^ Similar to the results from Kenya, interventions like this tended to focus on improving the supply side of health services and less of investment in promoting the demand side.^42^ As a result, our intervention had a limited impact on the prevention or population-level indicators. However, participating community members expressed appreciation for the emphasis on social accountability. We note the potential impact of such interventions with the eventual development of scalable models.^43^ For the purposes of the CODES trial, initial experiences with SMS reporting provided an affordable means of data collection. However, to optimise impact, online reporting platforms such as U-Report^44 45^ need to operate in conjunction with scalable accountability mechanisms. Community dialogue meetings may prove cost-prohibitive, if they require external facilitation.^10^ This area requires additional innovation and research, including an assessment of Uganda’s ‘*Barazas’* as a community forum for public debate on service delivery.

10.1136/bmjgh-2021-006084.supp7 Supplementary data

### Context, cost and policy

Several contextual factors influenced the implementation and effect of CODES. Health sector funding, particularly district-level budgets, remains a challenge. During the study period, health sector allocations fell from 8·7% of the national budget in 2013/2014 to 5·7% in 2015,^46^ despite the pre-existing national target of 15% by 2020. At the time, Uganda prioritised funding for infrastructure, energy and security over the ‘unproductive’ health sector. This trend, combined with the relatively high proportion of health expenditure allocated towards national hospitals (59·5% in 2015/2016) limited the fiscal and decision space available to district-level managers to implement primary health care (PHC) activities. Consequently, even as intervention districts acquired new managerial strategies, solutions remained unfunded and unimplemented due to a lack of discretionary resources. This severely limited the potential to solve health system problems locally, a key assumption underlying the CODES approach. This is in contrast to the situation in neighbouring Tanzania, where local district management teams have a discretionary fiscal space of 1 US$/capita.^47^

To offset Uganda’s limited fiscal space, UNICEF created a ‘bottleneck fund’ of US$10 000 per district to support the implementation of unfunded interventions, primarily on the supply side. In many cases, although relatively small, this was the only fungible resource, or ‘fiscal space’, available for bottleneck solutions. However, in some districts, the mere existence of a prioritised ‘bottleneck action plan’ enabled districts to leverage additional resources from other development partners in the country, which further emphasises the importance of the CODES approach.

A cost-effectiveness analysis for CODES will be published elsewhere. CODES was designed with the aim to be scaled up. We found that the costs of scale-up were low. scaling up the full package of CODES to all the districts of Uganda was only US$5 893 389. Findings of the analysis showed that technical assistance accounted for the largest proportion of the total costs (43% to 60% per year), followed by operational costs (17% to 27% per year), with the demand-side interventions taking up the least. These costs are low and can be absorbed by the MoH budget if it is increased slightly or increased by partners. The CODES project contributed to improved performance of district health systems in the eight wave 1 intervention districts, and if appropriately scaled up, it could improve the general performance of the national health system.

Thus, it is clear from the above that if districts are to implement evidence-informed implementation, they need commensurate fiscal and decision space. This, in turn, requires additional resources at the district level, with an increase in the proportion of domestic budgets allocated to the health sector. Furthermore, given that as much as half of the total health expenditure is derived from out-of-pocket expenditures in low-income countries, achieving quality coverage may require a dual focus on private and public healthcare providers.^41 48^

### Methodological considerations

This study employed customised LQAS surveys as the main data source for the bottleneck analysis. There are many advantages to the LQAS.^49^ However, during implementation of this study, we found that the use of LQAS can be cost prohibitive in Ugandan districts. In two times a day to scale the CODES intervention across Uganda, the government, therefore, now uses administrative data from district health information system-2 (DHIS2), which includes an automated function for graphing bottlenecks (the score card).^21^ This innovation requires further analysis to understand its utility and affordability and the implications of shifting from population to facility-based data.

The methodology for the CODES study presented certain limitations. The study relies on self-reported outcome data, as all household surveys. Furthermore, financial constraints prohibited a midterm coverage survey in comparison districts. The clustered design resulted in a larger than projected ICC of coefficients, leading to wide design-adjusted CIs and a possible failure to reach statistical significance for some indicators that otherwise showed positive effects from the CODES intervention.

While the findings are highly contextually dependent on local health systems, we suggest that the interventions may have similar effects in other decentralised health systems, with potential for larger effects where more resources and decision space are available to local managers.

## Conclusion

The CODES trial is the first of its kind to test whether district-level management interventions impact child health outcomes at the population level. Implementation of the CODES interventions led to modest increases in the effective coverage of curative care for malaria, pneumonia and diarrhoea symptoms in population-based surveys. The results indicate that the CODES approach should be considered in efforts to scale-up child health interventions across Uganda and in similar settings, and as a model for district health systems strengthening. Findings from the CODES study also point to the need for balance between demand and supply-side interventions, ensuring sufficient fiscal space and authority to local managers to act on findings, and the utility of implementation research to improve health system management.

10.1136/bmjgh-2021-006084.supp6 Supplementary data

## Data Availability

Data are available upon request. Data access for further research can be freely shared with researchers after a formal request through the corresponding author.
