# Supplementary material for: Child health and the implementation of Community and District-management Empowerment for Scale-up (CODES) in Uganda: a randomised controlled trial
Source: BMJ Glob Health. 2021 Jun 8;6(6):e006084. doi: 10.1136/bmjgh-2021-006084 (PMC8189926; doi:10.1136/bmjgh-2021-006084)
Supplement: Supplementary data [file bmjgh-2021-006084supp005.pdf]

Supplement Table 1: Characteristics of the 8 intervention and 8 comparison districts

Districts	Intervention/ Comparison	Year of establishment	Population (Total No. of People)*	DHMT size**	Stock*** ORS/Zn	Stock AMX	Stock ACT	Stock Vaccines	Essential Child Health Supplies ****
**Apac**	Intervention	1974	368786	Fully constituted	Y	Y	Y	Y	Y
**Arua**	Intervention	1980	785189	Not fully constituted	N	N	Y	Y	N
**Bugiri**	Intervention	1997	390076	Fully constituted	N	N	Y	Y	N
**Buhweju**	Intervention	2010	124044	Fully constituted	Y	Y	Y	N	N
**Buvuma**	Intervention	2010	89960	Not fully constituted	N	Y	Y	Y	N
**Luuka**	Intervention	2010	241453	Not fully constituted	N	N	N	N	Y
**Maracha**	Intervention	2010	186176	Fully constituted	N	N	N	N	N
**Masindi**	Intervention	1980	292951	Fully constituted	Y	Y	N	Y	N
**Alebtong**	Comparison	2010	225327	Not fully constituted	Y	N	Y	Y	Y
**Iganga**	Comparison	1980	506388	Fully constituted	N	N	N	N	N
**Kamuli**	Comparison	1980	490255	Fully constituted	N	N	N	Y	Y
**Kasese**	Comparison	1980	702029	Fully constituted	N	N	N	N	N
**Kiryandongo**	Comparison	2010	268188	Not fully constituted	N	N	Y	Y	N
**Kole**	Comparison	2010	241878	Not fully constituted	Y	N	Y	Y	Y
**Mitooma**	Comparison	2010	185519	Not fully constituted	Y	N	Y	Y	Y
**Sheema**	Comparison	2010	246636	Not fully constituted	Y	N	Y	Y	Y

*Population size
**Fully constituted
***Based on a decision rule for the number of health facilities with stock (Yes/No) in CODES Baseline Health Facility LQAS 2013 (Reference - CFI report Baseline Health Facility Assessment for Wave One Districts 2014)
****Based on a decision rule for the number of district health facilities with ALL essential supplies for child health on survey day (accessible and functional child scale, accessible and working infant scale, timer for counting respiratory rate, receptacle to administer ORS, MUAC tape) in CODES Baseline Health Facility LQAS 2013 – Note that none of the district health facilities complied with the decision rule for infrastructure (Reference- CFI Report Baseline Health Facility Assessment (HFA) for Wave One Districts 2014)
