# Supplementary material for: Child health and the implementation of Community and District-management Empowerment for Scale-up (CODES) in Uganda: a randomised controlled trial
Source: BMJ Glob Health. 2021 Jun 8;6(6):e006084. doi: 10.1136/bmjgh-2021-006084 (PMC8189926; doi:10.1136/bmjgh-2021-006084)

Supplemental Material 1: Graphics Summarizing CODES Theory of Change, Approach and Strategy

A: CODES implementation pillars

CODES INTERVENTION - RECAP
Community and District Empowerment for Scale-up

- The CODES Intervention aims to demonstrate that a **management strategy** based on three pillars of:
- 1) **improved targeting of interventions** to match disease burden, and **better allocation of resources** ;
- 2) **regular review and improvement of district health team performance**, and **use of evidence-based management tools** and **focal funding to overcome management bottlenecks**; and
- 3) **community oversight** and **inputs**, will lead to **improvements in both COVERAGE and QUALITY of key interventions** to reduce child deaths from diarrhoea, malaria and pneumonia.

B. CODES logic framework

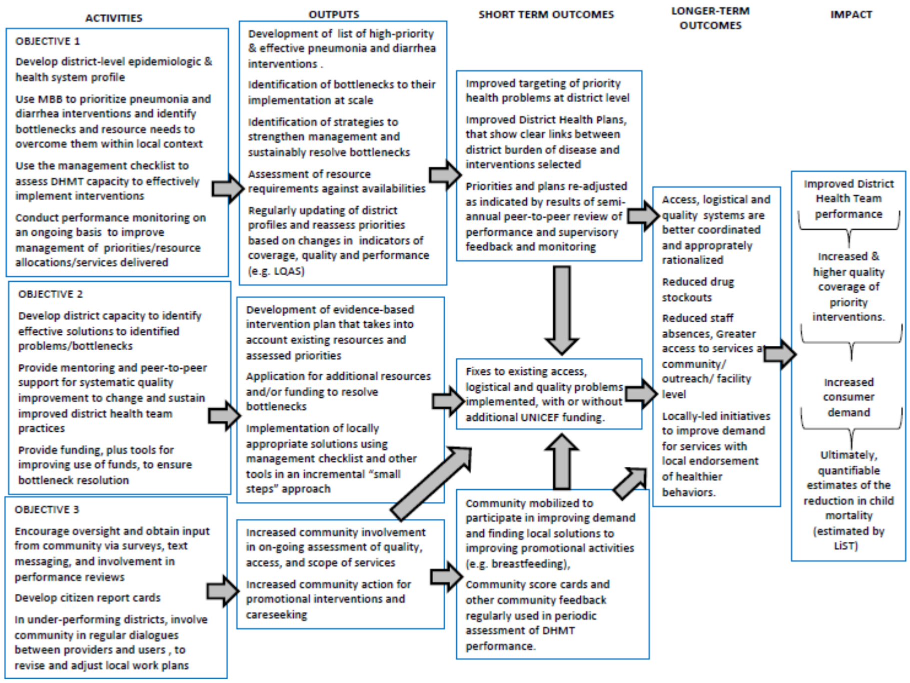

C. CODES district implementation research framework

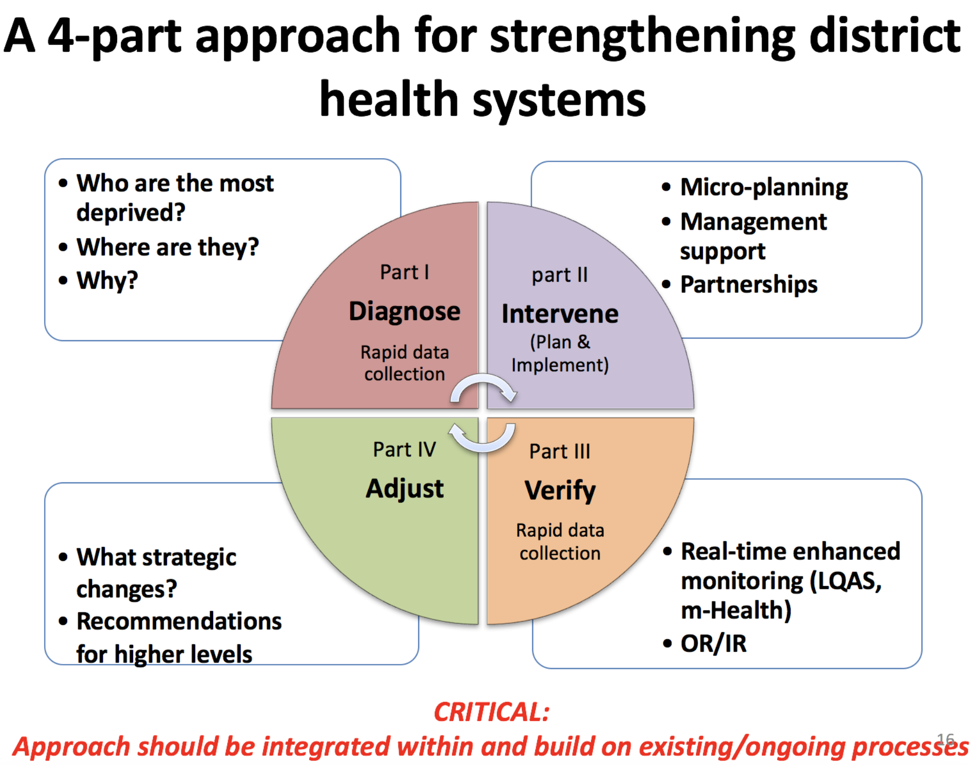

D. CODES implementation process framework for data acquisition and analysis

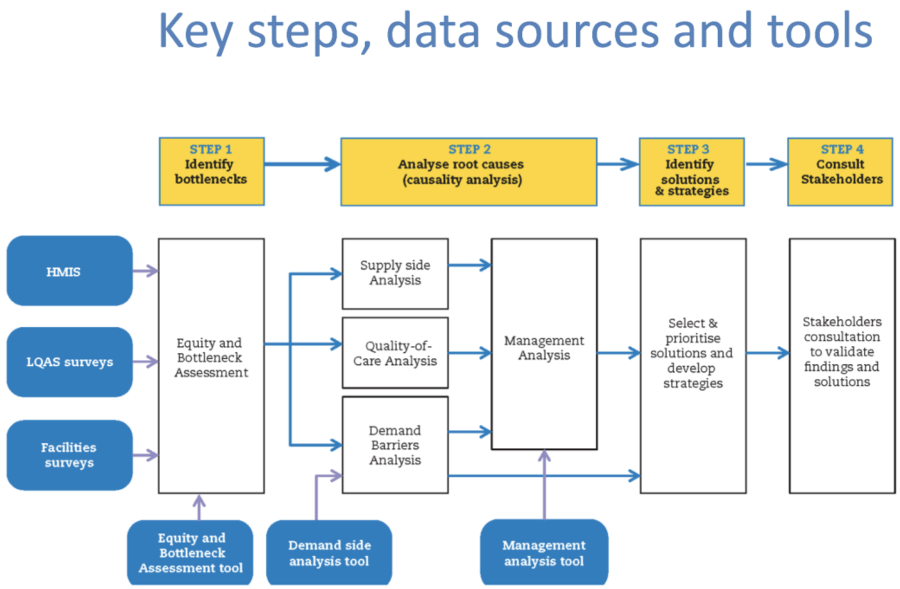

Supplement: Supplementary data [file bmjgh-2021-006084supp001.pdf]
