# Supplementary material for: Child health and the implementation of Community and District-management Empowerment for Scale-up (CODES) in Uganda: a randomised controlled trial
Source: BMJ Glob Health. 2021 Jun 8;6(6):e006084. doi: 10.1136/bmjgh-2021-006084 (PMC8189926; doi:10.1136/bmjgh-2021-006084)
Supplement: Supplementary data [file bmjgh-2021-006084supp002.pdf]

Supplement Figure 1: Citizen Report Cards

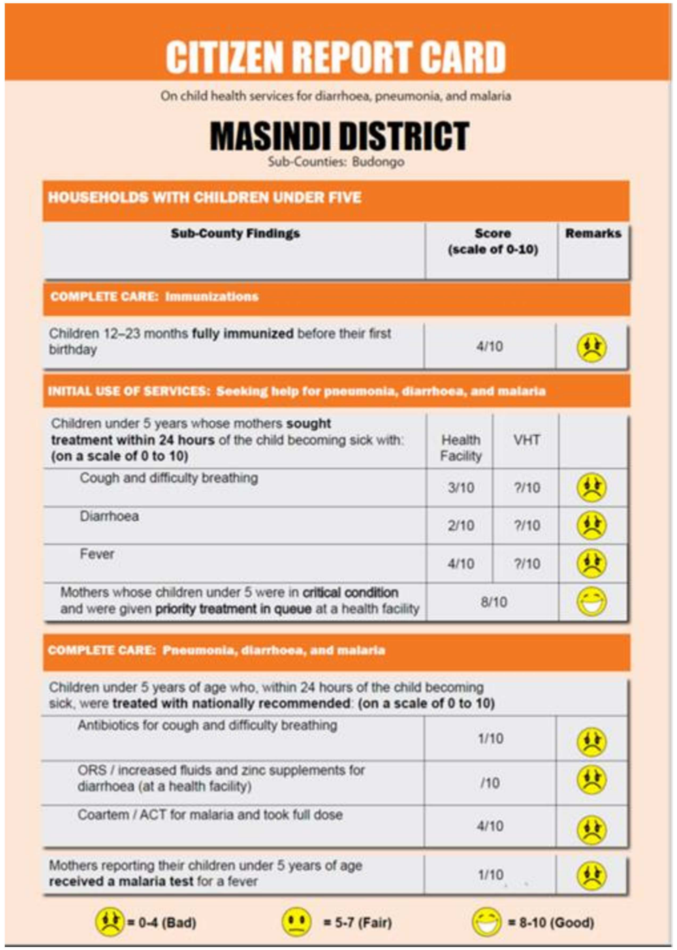

CITIZEN REPORT CARD

On child health services for diarrhoea, pneumonia, and malaria

MASINDI DISTRICT

Sub-Counties: Budongo

HOUSEHOLDS WITH CHILDREN UNDER FIVE			
**Sub-County Findings**	**Score (scale of 0-10)**		**Remarks**
**COMPLETE CARE: Immunizations**			
Children 12–23 months **fully immunized** before their first birthday	4/10		
**INITIAL USE OF SERVICES: Seeking help for pneumonia, diarrhoea, and malaria**			
Children under 5 years whose mothers **sought treatment within 24 hours** of the child becoming sick with: **(on a scale of 0 to 10)**	Health Facility	VHT	
Cough and difficulty breathing	3/10	?/10	
Diarrhoea	2/10	?/10	
Fever	4/10	?/10	
Mothers whose children under 5 were in **critical condition** and were given **priority treatment in queue** at a health facility	8/10		
**COMPLETE CARE: Pneumonia, diarrhoea, and malaria**			
Children under 5 years of age who, within 24 hours of the child becoming sick, were **treated with nationally recommended**: **(on a scale of 0 to 10)**			
Antibiotics for cough and difficulty breathing	1/10		
ORS / increased fluids and zinc supplements for diarrhoea (at a health facility)	/10		
Coartem / ACT for malaria and took full dose	4/10		
Mothers reporting their children under 5 years of age **received a malaria test** for a fever	1/10		

= 0-4 (Bad) = 5-7 (Fair) = 8-10 (Good)

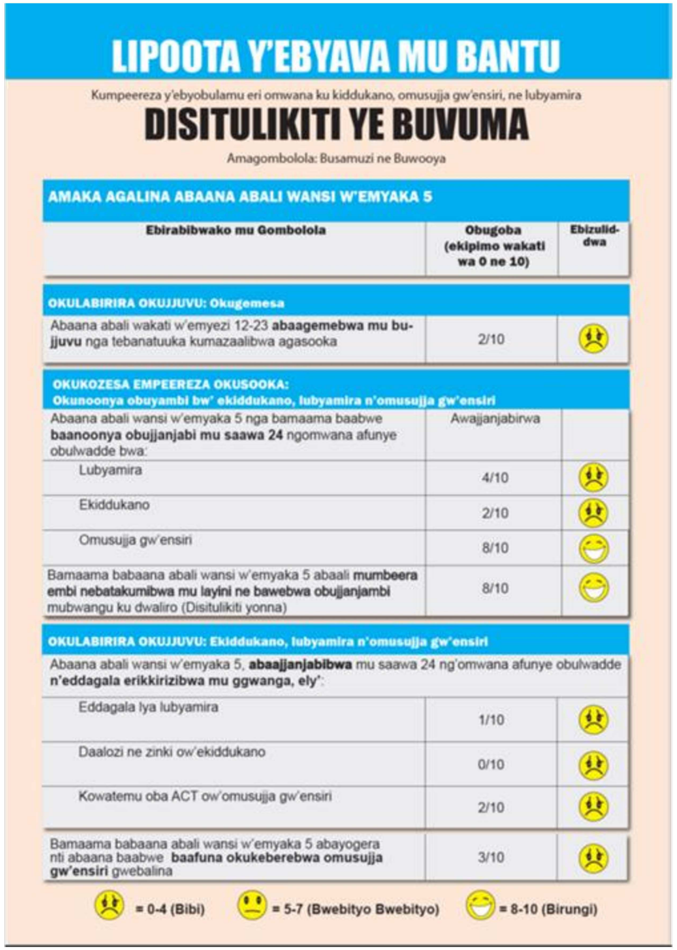

LIPOOTA Y'EBYAVA MU BANTU

Kumpeereza y'ebyobulamu eri omwana ku kiddukano, omusujja gw'ensiri, ne lubyamira

DISITULIKITI YE BUVUMA

Amagombolola: Busamuzi ne Buwooya

AMAKA AGALINA ABAANA ABALI WANSI W'EMYAKA 5		
**Ebirabibwako mu Gombolola**	**Obugoba (ekipimo wakati wa 0 ne 10)**	**Ebizulid-dwa**
**OKULABIRIRA OKUJJUVU: Okugemesa**		
Abaana abali wakati w'emyezi 12-23 **abaagemebwa mu bu-jjuvu** nga tebanatuuka kumazaalibwa agasooka	2/10	
**OKUKOZESA EMPEEREZA OKUSOOKA: Okunoonya obuyambi bw' ekiddukano, lubyamira n'omusujja gw'ensiri**		
Abaana abali wansi w'emyaka 5 nga bamaama baabwe **baanoonya obujjanjabi mu saawa 24** ngomwana afunye obulwadde bwa:	Awajjanjabirwa	
Lubyamira	4/10	
Ekiddukano	2/10	
Omusujja gw'ensiri	8/10	
Bamaama babaana abali wansi w'emyaka 5 abaali **mumbeera embi nebatakumibwa mu layini ne bawebwa obujjanjambi** mubwangu ku dwaliro (Disitulikiti yonna)	8/10	
**OKULABIRIRA OKUJJUVU: Ekiddukano, lubyamira n'omusujja gw'ensiri**		
Abaana abali wansi w'emyaka 5, **abaajjanjabibwa** mu saawa 24 ng'omwana afunye obulwadde **n'eddagala erikkirizibwa mu ggwanga, ely'**:		
Eddagala lya lubyamira	1/10	
Daalozi ne zinki ow'ekiddukano	0/10	
Kowatemu oba ACT ow'omusujja gw'ensiri	2/10	
Bamaama babaana abali wansi w'emyaka 5 abayogera nti abaana baabwe **baafuna okukeberebwa omusujja gw'ensiri** gwebalina	3/10	

= 0-4 (Bibi) = 5-7 (Bwebityo Bwebityo) = 8-10 (Birungi)
