# Supplementary figures and images for: Child health and the implementation of Community and District-management Empowerment for Scale-up (CODES) in Uganda: a randomised controlled trial
Source: BMJ Glob Health. 2021 Jun 8;6(6):e006084. doi: 10.1136/bmjgh-2021-006084 (PMC8189926; doi:10.1136/bmjgh-2021-006084)

**Supplement Figure 2: Consideration for stratification**

# Wave 1 – Assignment Overview

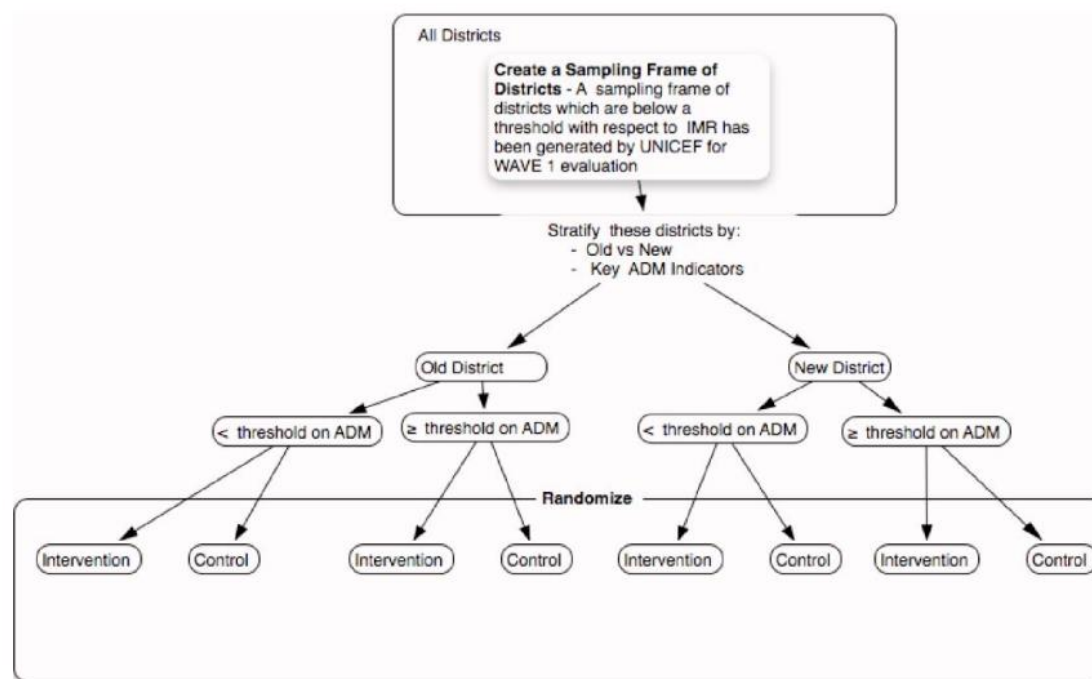

Supplement: Supplementary data [file bmjgh-2021-006084supp003.pdf]

# WAVE 1 – Sampling Frame

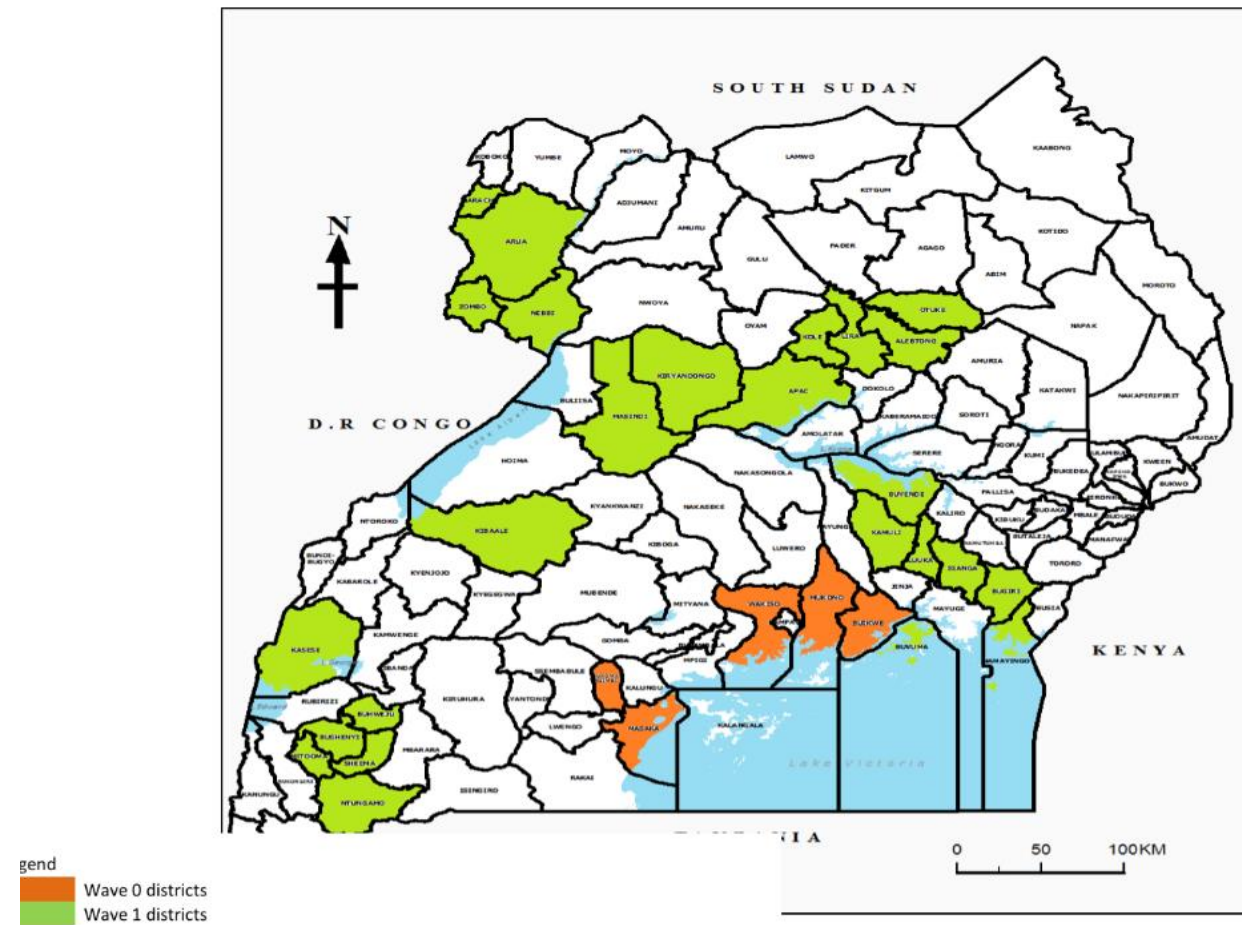

Supplement: Supplementary data [file bmjgh-2021-006084supp004.pdf]
