# Supplementary material for: Child health and the implementation of Community and District-management Empowerment for Scale-up (CODES) in Uganda: a randomised controlled trial
Source: BMJ Glob Health. 2021 Jun 8;6(6):e006084. doi: 10.1136/bmjgh-2021-006084 (PMC8189926; doi:10.1136/bmjgh-2021-006084)
Supplement: Supplementary data [file bmjgh-2021-006084supp008.pdf]

Supplement Table 2: Socio-demographic characteristics

	Observed % (95% CI)			Difference in differences (DID)		
	Intervention	Comparison	P-value*	DID change in %	95 % CI	P-value*
**Households with children (0-59 months)**						
**Baseline**	n=663	n=851				
**Endline**	n=761	n=757				
**Demographic**						
**Mother's age**						
**Baseline**	n=665	n=847				
**Endline**	n=753	n=752				
**Baseline (mean years)**	29·5 (28.9-30.1)	30·7 (29.9-31.5)	0.029			
**Endline (mean years)**	29.0 (28.3-29.7)	29·7 (29.1-30.3)	0.14	0·45	-0·57,+1·47	0·363
**Marital status (married*)**						
**Baseline**	n=662	n=850				
**Endline**	n=758	n=754				
**Baseline**	87.9 (84.5-90.7)	88.5 (85.8-90.7)	0.78			
**Endline**	89.1 (86.1-91.5)	85.8 (80.8-89.7)	0.19	3.8	-3.1,+10.7	0.261
**Socio-economic**						
**Household of a child aged 0-59 months with roofing (asbestos, iron sheets, tiles)**						
**Baseline**	n-663	n=851				
**Endline**	n=761	n=757				
**Baseline**	47·8 (35.9-60.0)	66.0 (54.2-76.2)	0.034			
**Endline**	59·8 (48.6-70.0)	67·1 (55.2-77.2)	0.35	10·9	4·2,17·6	0·003
**Household of a child aged 0-59 months with electricity**						
**Baseline**	5·7 (3.2-10.0)	9·6 (5.8-15.5)	0.16			
**Endline**	21·2 (16.6-26.5)	23·1 (18.6-28.3)	0.58	1·9	-10.0,+13.9	0·734
**Mothers inability to read**						
**Baseline**	n=663	n=851				
**Endline**	n=761	n=757				

**Baseline**	40.0 (35.2-45.0)	38.3 (33.3-43.6)	0.65			
**Endline**	40.3 (35.8-45.1)	37.0 (31.7-42.6)	0.36	1.7	-8.0,+11.3	0.714
**Household has Radio**						
**Baseline**	62.0 (57.2-66.5)	67.1 (62.4-71.5)	0.12			
**Endline**	54.0 (48.6-59.4)	58.7 (54.7-62.5)	0.17	0.5	-9.3,+10.2	0.921

*Study design adjusted
**Includes co-habiting
