# Supplementary material for: Child health and the implementation of Community and District-management Empowerment for Scale-up (CODES) in Uganda: a randomised controlled trial
Source: BMJ Glob Health. 2021 Jun 8;6(6):e006084. doi: 10.1136/bmjgh-2021-006084 (PMC8189926; doi:10.1136/bmjgh-2021-006084)
Supplement: Supplementary data [file bmjgh-2021-006084supp007.pdf]

Supplement Table 4: Coverage indicators

	Coverage indicator
**Protect**	
**Acute Respiratory Infection (ARI)**	
	Consumption of Vitamin A amongst children aged 12-23 months in the last 6months
	Consumption of Vitamin A amongst children aged 6-11 months in the last 6 months
	Exclusive breastfeeding for the first five months amongst children aged 6-11 months
**General**	
	Mothers of children aged 0-5 months who attended ANC at least 4 times during last pregnancy
	Mothers of children aged 0-59 months who gave birth in a heath facility with the assistance of a skilled birth attendant during last pregnancy
	Mothers of children aged 0-5 months who received HIV counselling, testing and notification of results during the last pregnancy
**Prevent**	
**ARI /Pneumonia**	Children aged 12-23 months without verified DPT3
	Children aged 12-23 months with verified DPT3
	Children aged 12-23 months fully vaccinated (verified for BCG, polio 4+, DPT3+ and measles)
	Children aged 12-23 months vaccinated for polio <2 weeks, DPT and measles (verified for BCG, polio 4+, DPT3+ and measles)
**Malaria**	
	Children aged 0-59 months who slept under an ITN the night prior to the survey
	Children aged 0-59 months who used a verified LLN (without holes) the night prior to the survey
**Diarrhea (Safe Water/Sanitation/Handwashing)**	
	Children aged 0-59 months in households with an improved water source (piped , protected well or bore hole)
	Children aged 0-59 months in households with observed covered water containers
	Children aged 0-59 months in households equipped with observed latrine/toilet, complete with super structure and cover
	Children aged 0-59 months whose last stool was disposed of in a latrine (i.e. safely)
	Children aged 0-59 months whose mothers used soap to hand wash at a minimum of two critical junctures in the 24 hours prior to the survey (after defecating; after cleaning a child who had defecated; before preparing a meal; or before feeding a child)
**Treatment (timely & appropriate case management)**	
**ARI/Pneumonia**	
	Children aged 0-59 months with pneumonia who received the full course of recommended amoxicillin-based treatment
**Malaria**	
	Children aged 0-59 months with confirmed malaria who received the full course of recommended ACT-based treatment
**Diarrhea**	
	Children aged 0-59 months with diarrhea (3+ watery or bloody stools on the same day) in the last two weeks who received the full course of recommended zinc and ORS treatment

	Coverage indicator
	Children aged 0-59 months with diarrhea (3+ watery or bloody stools on the same day) in the last two weeks who received the full course of recommended zinc and ORS treatment (starting on the first day and lasting the recommended duration)
	Children aged 0-59 months with diarrhea (3+ watery or bloody stools on the same day) in the last two weeks who received any amount of the recommended zinc and ORS treatment
**Prevalence**	
	Households reporting 2 week recall of fever in resident children aged 0-59 months
	Households reporting 2 week recall of cough in resident children aged 0-59 months
	Households reporting 2 week recall of diarrhea in resident children aged 0-59 months
