# Supplementary material for: Child health and the implementation of Community and District-management Empowerment for Scale-up (CODES) in Uganda: a randomised controlled trial
Source: BMJ Glob Health. 2021 Jun 8;6(6):e006084. doi: 10.1136/bmjgh-2021-006084 (PMC8189926; doi:10.1136/bmjgh-2021-006084)
Supplement: Supplementary data [file bmjgh-2021-006084supp006.pdf]

Supplement Table 3: Implementation of the Three Pillars of CODES in Each Implementing District (i.e. Dosage and timing of implementation)

Pillar of intervention	Apac	Arua	Bugiri	Buweju	Buvuma	Luuka	Maracha	Masindi
**Pillar 1***								
LQAS data collection baseline*	09/12/2013	09/12/2013	09/12/2013	09/12/2013	09/12/2013	09/12/2013	09/12/2013	09/12/2013
LQAS data collection mid-term*	06/11/2014<br>20/11/2015	06/11/2014<br>20/11/2015	06/11/2014<br>20/11/2015	06/11/2014<br>20/11/2015	06/11/2014<br>20/11/2015	06/11/2014<br>20/11/2015	06/11/2014<br>20/11/2015	06/11/2014<br>20/11/2015
LQAS data collection endline*	09/30/2016	09/30/2016	09/30/2016	09/30/2016	09/30/2016	09/30/2016	09/30/2016	09/30/2016
Use of Tanahashi model to identify bottlenecks*	27/01/2014<br>04/02/2015<br>10/02/2016	27/01/2014<br>04/02/2015<br>10/02/2016	27/01/2014<br>04/02/2015<br>10/02/2016	27/01/2014<br>04/02/2015<br>10/02/2016	27/01/2014<br>04/02/2015<br>10/02/2016	27/01/2014<br>04/02/2015<br>10/02/2016	27/01/2014<br>04/02/2015<br>10/02/2016	27/01/2014<br>04/02/2015<br>10/02/2016
Causal Analysis to identify root causes*	27/01/2014<br>04/02/2015<br>10/02/2016	27/01/2014 ,<br>04/02/2015<br>10/02/2016	27/01/2014<br>04/02/2015<br>10/02/2016	27/01/2014<br>04/02/2015<br>10/02/2016	27/01/2014<br>04/02/2015<br>10/02/2016	27/01/2014<br>04/02/2015<br>10/02/2016	27/01/2014<br>04/02/2015<br>10/02/2016	27/01/2014<br>04/02/2015<br>10/02/2016
Other LQAS activities outside of CODES (specify)	N	N	N	N	N	N	N	N
**Pillar 2**								
Training on the use of management tools	27/01/2014	27/01/2014	27/01/2014	27/01/2014	27/01/2014	27/01/2014	27/01/2014	27/01/2014
Completed causal analysis, based on management checklist*	27/01/2014<br>04/02/2015<br>10/02/2016	27/01/2014<br>04/02/2015<br>10/02/2016	27/01/2014<br>04/02/2015<br>10/02/2016	27/01/2014<br>04/02/2015<br>10/02/2016	27/01/2014<br>04/02/2015<br>10/02/2016	27/01/2014<br>04/02/2015<br>10/02/2016	27/01/2014<br>04/02/2015<br>10/02/2016	27/01/2014<br>04/02/2015<br>10/02/2016
Documented peer-to - peer mentoring process*	11/02/2013<br>10/12/2014<br>28/10/2015<br>01/11/2016	11/02/2013<br>10/12/2014<br>28/10/2015<br>01/11/2016	11/02/2013<br>10/12/2014<br>28/10/2015<br>01/11/2016	11/02/2013<br>10/12/2014<br>28/10/2015<br>01/11/2016	11/02/2013<br>10/12/2014<br>28/10/2015<br>01/11/2016	11/02/2013<br>10/12/2014<br>28/10/2015<br>01/11/2016	11/02/2013<br>10/12/2014<br>28/10/2015<br>01/11/2016	11/02/2013<br>10/12/2014<br>28/10/2015<br>01/11/2016
Documented annual performance assessments* (Development of annual	Y	Y	Y	Y	Y	Y	Y	Y

**Pillar of intervention**	**Apac**	**Arua**	**Bugiri**	**Buweju**	**Buvuma**	**Luuka**	**Maracha**	**Masindi**
scorecards)								
Documented situational analysis to identify a) low coverage areas; b) underserved populations; and c) bottlenecks*	Y	Y	Y	Y	Y	Y	Y	Y
Documented evidence of CQI activity independent of CODES (specify)	Y – Comprehensive HFQAP	Y – Comprehensive HFQAP	Y – Comprehensive HFQAP	Y – Comprehensive HFQAP	Y – Comprehensive HFQAP	Y – Comprehensive HFQAP	Y – Comprehensive HFQAP	Y – Comprehensive HFQAP
**Pillar 3****								
Community dialogues (number)**	17	17	17	17	17	17	17	17
Documented use of citizen report cards**	Y (Annually)	Y (Annually)	Y (Annually)	Y (Annually)	Y (Annually)	Y (Annually)	Y (Annually)	Y (Annually)
Documented use of text message surveys**	Y(2 rounds annually)	Y(2 rounds annually)	Y(2 rounds annually)	Y(2 rounds annually)	Y(2 rounds annually)	Y(2 rounds annually)	Y(2 rounds annually)	Y(2 rounds annually)
Community score card developed**	N	N	N	N	N	N	N	N
Documented use of U-Report**	Y(2 polls per year)	Y(2 polls per year)	Y(2 polls per year)	Y(2 polls per year)	Y(2 polls per year)	Y(2 polls per year)	Y(2 polls per year)	Y(2 polls per year)
Documented use of Rapid-SMS**	N	N	N	N	N	N	N	N
Documented use of supplemental non-CODES community-based mechanisms (specify)	Y- Policy meetings and involvement of political leadership	Y- Policy meetings and involvement of political leadership	Y- Policy meetings and involvement of political leadership	Y- Policy meetings and involvement of political leadership	Y- Policy meetings and involvement of political leadership	Y- Policy meetings and involvement of political leadership	Y- Policy meetings and involvement of political leadership	Y- Policy meetings and involvement of political leadership

Pillar of intervention	Apac	Arua	Bugiri	Buweju	Buvuma	Luuka	Maracha	Masindi
Involvement of additional community-based organizations (CBOs), external to the CODES trial (specify)	Y (3 CBOs identified)	Y (3 CBOs identified)	Y (3 CBOs identified)	Y (3 CBOs identified)	Y (3 CBOs identified)	Y (3 CBOs identified)	Y (3 CBOs identified)	Y (3 CBOs identified)

*Source: CFI Reports and work plan reports
** Source: ACODE Reports and submitted work plan reports
Study findings were disseminated through three national meetings, one written research brief, and one written policy brief. In addition, five of eight planned manuscripts are published in peer-reviewed journals.
